# Supplementary material for: Off-pump versus on-pump coronary artery surgery in octogenarians (from the KROK Registry)
Source: PLoS One. 2020 Sep 10;15(9):e0238880. doi: 10.1371/journal.pone.0238880 (PMC7482977; doi:10.1371/journal.pone.0238880)
Supplement: Attachment — Submitted filename: Response to Reviewers_3.doc [file pone.0238880.s005.doc]

PONE-D-20-11113R1
Off-pump versus on-pump coronary artery surgery in octogenarians (from the KROK Registry)
PLOS ONE

Dear Dr. Knapik,

Thank you for submitting your manuscript to PLOS ONE. After careful consideration, we feel that it has merit but does not fully meet PLOS ONE’s publication criteria as it currently stands. Therefore, we invite you to submit a revised version of the manuscript that addresses the points raised during the review process.

Please submit your revised manuscript by Sep 12 2020 11:59PM. If you will need more time than this to complete your revisions, please reply to this message or contact the journal office at [plosone@plos.org](mailto:plosone@plos.org). When you're ready to submit your revision, log on to <https://www.editorialmanager.com/pone/> and select the 'Submissions Needing Revision' folder to locate your manuscript file.

Please include the following items when submitting your revised manuscript:

- A rebuttal letter that responds to each point raised by the academic editor and reviewer(s). You should upload this letter as a separate file labeled 'Response to Reviewers'.
- A marked-up copy of your manuscript that highlights changes made to the original version. You should upload this as a separate file labeled 'Revised Manuscript with Track Changes'.
- An unmarked version of your revised paper without tracked changes. You should upload this as a separate file labeled 'Manuscript'.

If you would like to make changes to your financial disclosure, please include your updated statement in your cover letter. Guidelines for resubmitting your figure files are available below the reviewer comments at the end of this letter.

If applicable, we recommend that you deposit your laboratory protocols in protocols.io to enhance the reproducibility of your results. Protocols.io assigns your protocol its own identifier (DOI) so that it can be cited independently in the future. For instructions see: [http://journals.plos.org/plosone/s/submission-guidelines#loc-laboratory-protocols](http://journals.plos.org/plosone/s/submission-guidelines#loc-laboratory-protocols)

We look forward to receiving your revised manuscript.

Kind regards,

Mariusz Kowalewski
Academic Editor
PLOS ONE

**Additional Editor Comments (if provided):**

**We are interested in publishing your manuscript; however, one reviewer continues to have significant concerns with your revised manuscript. Before the paper can be accepted in its final form we would like your comments to the critiques. Accordingly, we invite you to respond to all the reviewers' comments and recommendations. A decision on acceptability of your manuscript will be made only after the revised version has been reevaluated.

In particular, the Reviewer is concerned about exclusion of conversion cases and censoring the time-to-event analysis by the date of discharge. While removing in-hospital mortality from the analysis may better help understand the postoperative sequelae in these patients unobscured by features inherent to the in-hospital course, the principal analysis should include both in-hospital and long-term; i therefore suggest to keep censored analysis to the supplement only but cite the results in the respective paragraph, similarly for the conversion cases, please report the mortality rates for both per-protocol and ITT scenarios.**

**Answer:**

As previously explained, 53 conversions were identified among 6,006 patients (0.9%), however the type of surgical procedure was marked differently in these patients. For patients with conversion, users of the KROK database chose either the finally performed surgical procedure (CABG) or the originally planned surgical procedure (OPCAB). Therefore, to avoid any doubt with the grouping variable (OPCAB/CABG), we proposed to exclude these patients. The number of patients was therefore reduced, and their total number was reduced from 6,006 to 5,953 patients.

We understand however, that the Reviewer might have been concerned about exclusion of conversion cases, as the in-hospital mortality in this subgroup of 53 patients was as high as 32%. Therefore, we decided to follow the intention-to-treat approach and all patients with conversion were allocated in the OPCAB group. In the Results section however, we decided to present mortality rates for both per-protocol and ITT scenario.

Additionally, the Reviewer was also concerned about censoring the time-to-event analysis by the date of discharge, and indicated that the principal analysis should include both in-hospital and long-term results.

While we generally agree with this view, we feel that using the day of operation as a starting point might create a lot of bias in this particular situation. The reasons for that have been already explained. In case of significant in-hospital mortality and relatively frequent postoperative complications (among our octogenarians undergoing CABG), this would create an influence of in-hospital mortality on long-term results. The survival curves would in this case, separate at the beginning and then remain parallel during the whole postoperative course (even when in fact there was no difference in the long-term results). It turned out, that we were right – when the starting point of our analysis was changed, the survival curves following propensity scoring starting at the day of operation indicated that long-term survival was significantly better (p=0.013) in the OPCAB group (despite the fact that all conversions are being now analyzed in the OPCAB group).

We understand however, that the Reviewer might have been concerned with our previous approach. Therefore, we performed the principal analysis starting at the day of the operation and took Editors’ comment to keep censored analysis to the supplement only.

**Action:**

All patients with conversion were allocated in the OPCAB group. As a consequence, all results had to be recalculated and new data are now presented in all tables and figures, and appropriate changes were also made in the whole text of the manuscript. Additionally, in the Results section, we presented mortality rates for both per-protocol and ITT scenario, as required.

Also, we performed the principal analysis starting at the day of the operation. Additionally, we presented our censored analysis to the supplement only (as Supplementary file 3), presenting the results of this additional analysis in the Results section. On a basis of these calculations, we had to change one of the results of our study (regarding a long-term follow up).

Reviewers' comments:

Reviewer's Responses to Questions

**Comments to the Author**

1. If the authors have adequately addressed your comments raised in a previous round of review and you feel that this manuscript is now acceptable for publication, you may indicate that here to bypass the “Comments to the Author” section, enter your conflict of interest statement in the “Confidential to Editor” section, and submit your "Accept" recommendation.

Reviewer #1: All comments have been addressed

Reviewer #3: (No Response)

Reviewer #4: All comments have been addressed

2. Is the manuscript technically sound, and do the data support the conclusions?

The manuscript must describe a technically sound piece of scientific research with data that supports the conclusions. Experiments must have been conducted rigorously, with appropriate controls, replication, and sample sizes. The conclusions must be drawn appropriately based on the data presented.

Reviewer #1: Yes

Reviewer #3: No

Reviewer #4: (No Response)

3. Has the statistical analysis been performed appropriately and rigorously?

Reviewer #1: Yes

Reviewer #3: Yes

Reviewer #4: (No Response)

4. Have the authors made all data underlying the findings in their manuscript fully available?

The [PLOS Data policy](http://www.plosone.org/static/policies.action#sharing) requires authors to make all data underlying the findings described in their manuscript fully available without restriction, with rare exception (please refer to the Data Availability Statement in the manuscript PDF file). The data should be provided as part of the manuscript or its supporting information, or deposited to a public repository. For example, in addition to summary statistics, the data points behind means, medians and variance measures should be available. If there are restrictions on publicly sharing data—e.g. participant privacy or use of data from a third party—those must be specified.

Reviewer #1: Yes

Reviewer #3: Yes

Reviewer #4: (No Response)

5. Is the manuscript presented in an intelligible fashion and written in standard English?

PLOS ONE does not copyedit accepted manuscripts, so the language in submitted articles must be clear, correct, and unambiguous. Any typographical or grammatical errors should be corrected at revision, so please note any specific errors here.

Reviewer #1: Yes

Reviewer #3: Yes

Reviewer #4: (No Response)

**6. Review Comments to the Author.**

**Please use the space provided to explain your answers to the questions above. You may also include additional comments for the author, including concerns about dual publication, research ethics, or publication ethics. (Please upload your review as an attachment if it exceeds 20,000 characters)**

**Reviewer #1:**

**The authors adequately addressed all of the concerns. Nevertheless, there remains a certain risk of bias due to multiple confounders. Therefore, the authors should consider to tune down their conclusion.**

**Answer:**

This issue has been already raised in a previous round by another Reviewer who stated: „Your conclusion paragraph states a little too strongly that OPCAB should be the method of choice in octogenarians and this statement and the data may not apply to certain subsets of patient where on pump CABG would be a better choice. This statement should be softened…”

On a basis of this comment, we have decided to change the conclusion according to the Reviewer’s suggestion. Therefore, the conclusion has been already tuned down during a previous review round.

**Action:**

At the end of the Discussion section, the following sentence replaced the previous statement: “Based on the results of this study, we suggest that the OPCAB technique should be considered as perfectly acceptable in octogenarians when performed by surgeons experienced in the technique”.

Also, in the conclusion of the abstract, the sentence has been changed to: “On the basis of our findings we suggest that off pump technique should be considered as perfectly acceptable in octogenarians.”

These changes have been already done during a previous review round.

**Reviewer #3:**

**1. The elimination of the conversion patients (which should be almost all OPCAB converted to CABG since many fewer might be converted to OPCAB from CABG due to porcelain aorta, etc. I do not believe that these patients should be excluded because they have a very high mortality rate which therefore biases the results if these were intended to be OPCAB patients!**

**Answer:**

As explained during a previous review round, 53 conversions were identified among 6,006 patients (0.9%), however the type of surgical procedure was marked differently in these patients. For patients with conversion, users of the KROK database chose either the finally performed surgical procedure (CABG) or the originally planned surgical procedure (OPCAB). Therefore, to avoid any doubt with the grouping variable (OPCAB/CABG), we initially proposed to exclude these patients. The number of patients was therefore reduced, and their total number was reduced from 6,006 to 5,953 patients in a previous version of the manuscript.

We understand however, that exclusion of conversion cases might not be acceptable, particularly when the in-hospital mortality in this subgroup of 53 patients was as high as 32%. Therefore, we decided to follow the intention-to-treat approach and all patients with conversion were allocated in the OPCAB group. In the Results section however, we decided to present mortality rates for both per-protocol and ITT scenario, as required.

**Action:**

All patients with conversion were allocated in the OPCAB group. As a consequence, all results had to be recalculated and new data are now presented in all tables and figures, and appropriate changes were also made in the whole text of the manuscript. Additionally, in the Results section, we presented mortality rates for both per-protocol and ITT scenario (as proposed by the Editor).

In the Results section the following sentence was therefore added: “Conversions from OPCAB do CABG were analyzed in the OPCAB group”.

**2. The authors explanation for the bias introduced by using the date of discharge as the start of survival is unsupportable. The elimination of mortality by this method cannot be avoided by surgeons and therefore to say that "if you make it to discharge" that OPCAB is more likely to survive (but the opposite is true if you take into account the mortality of conversions and perioperative deaths) is erroneous and not acceptable.**

**Answer:**

We understand, that the Reviewer might have been concerned with our approach, when the survival curves start at the date of discharge. Therefore, we now performed the principal analysis starting at the day of the operation and took Editors’ comment to keep censored analysis to the supplement only, presenting the results of this additional analysis in the respective paragraph.

**Action:**

We recalculated our data and performed the principal analysis starting at the day of the operation. Additionally, we presented our censored analysis to the supplement only (adding Supplementary file 3), presenting the results of this additional analysis in the Results section. Changes were also done in the whole text of the manuscript, as appropriate.

When the starting point of our analysis was changed, the survival curves following propensity scoring starting at the day of operation indicated that long-term survival was significantly better (p=0.013) in the OPCAB group (despite the fact that all conversions are being now analyzed in the OPCAB group). Therefore, we had to do some minor changes in the text of the manuscript (in the abstract, and in the Results and Discussion section)..

Reviewer #4: (No Response)

7. PLOS authors have the option to publish the peer review history of their article ([what does this mean?](https://journals.plos.org/plosone/s/editorial-and-peer-review-process#loc-peer-review-history)). If published, this will include your full peer review and any attached files.


If you choose “no”, your identity will remain anonymous but your review may still be made public.


**Do you want your identity to be public for this peer review?** For information about this choice, including consent withdrawal, please see our [Privacy Policy](https://www.plos.org/privacy-policy).

Reviewer #1: No

Reviewer #3: No

Reviewer #4: No

[NOTE: If reviewer comments were submitted as an attachment file, they will be attached to this email and accessible via the submission site. Please log into your account, locate the manuscript record, and check for the action link "View Attachments". If this link does not appear, there are no attachment files.]

While revising your submission, please upload your figure files to the Preflight Analysis and Conversion Engine (PACE) digital diagnostic tool, <https://pacev2.apexcovantage.com/>. PACE helps ensure that figures meet PLOS requirements. To use PACE, you must first register as a user. Registration is free. Then, login and navigate to the UPLOAD tab, where you will find detailed instructions on how to use the tool. If you encounter any issues or have any questions when using PACE, please email PLOS at [figures@plos.org](mailto:figures@plos.org). Please note that Supporting Information files do not need this step.
